# LPS-Induced Liver Inflammation Is Inhibited by Psilocybin and Eugenol in Mice

**DOI:** 10.3390/ph18040451

**Published:** 2025-03-23

**Authors:** Gregory Ian Robinson, Marta Gerasymchuk, Timur Zanikov, Esmaeel Ghasemi Gojani, Shima Asghari, Alyssa Groves, Lucie Haselhorst, Sanjana Nandakumar, Cora Stahl, Ceejay Cruz, Mackenzie Cameron, Yeva Zahoruiko, Dongping Li, Rocio Rodriguez-Juarez, Alex Snelling, Darryl Hudson, Anna Fiselier, Olga Kovalchuk, Igor Kovalchuk

**Affiliations:** 1 Department of Biological Sciences, University of Lethbridge, Lethbridge, AB T1K 3M4, Canadamarta.gerasymchuk@uleth.ca (M.G.);; 2 Cumming School of Medicine, University of Calgary, Calgary, AB T2N 4N1, Canada; 3 Institute for Medical Nutrition Science, Universität zu Lübeck, 23562 Lübeck, Germany; 4 School of Biosciences and Technology, Vellore Institute of Technology, Vellore 632014, India; 5 Department of Medicine, Medical Sciences, and Nutrition, King’s College, University of Aberdeen, Aberdeen AB24 3FX, UK; 6 GoodCap Pharmaceuticals, 520 3rd Avenue SW, Suite 1900, Calgary, AB T2P 0R3, Canada

**Keywords:** psilocybin, eugenol, psychedelics, inflammation, lipopolysaccharide, liver

## Abstract

**Background/Objectives:** Liver inflammatory diseases are a major global health burden and are often exacerbated by inflammation driven by lipopolysaccharides (LPS) through toll-like receptor 4 signaling. This study evaluates the anti-inflammatory effects of psilocybin and eugenol in an LPS-induced liver inflammation model in C57BL/6J mice. **Methods:** Mice were treated with psilocybin (0.88 mg/kg) and/or eugenol (17.59 mg/kg) either before (pre-treatment) or after (post-treatment) LPS injection. **Results:** Psilocybin and eugenol, individually and in combination, significantly reduced the LPS-induced mRNA levels of pro-inflammatory cytokines, with post-treatment administration exhibiting stronger effects than pre-treatment. Psilocybin alone displayed the most pronounced anti-inflammatory response, especially for *IL-1β*, *IL-6*, and *MCP-1*, while its combination with eugenol in 1:50 ratio demonstrated similar results, with strongly reduced *COX-2* and *TNF-α*. Histological analysis revealed improved nuclear circularity and reduced inflammatory infiltration in the treatment groups. Eugenol alone showed potential adverse effects, including increased *MCP-1* and *GM-CSF*, but this was mitigated by the co-administration of psilocybin. **Conclusions:** These findings highlight psilocybin and its combination with eugenol as promising therapies for hepatic inflammation, suggesting their application in treating acute and chronic liver diseases. Future research should explore their long-term effects, the mechanisms underlying their anti-inflammatory actions, and their therapeutic efficacy in humans.

## 1. Introduction

Liver diseases are a growing public health concern, causing approximately 2 million deaths worldwide per year [1]. The etiology of liver diseases is diverse; however, hepatic inflammation acts as a pathogenic mechanism that is linked to disease severity in multiple hepatic pathological states, including non-alcoholic fatty liver disease (NAFLD), hepatitis, and cirrhosis [2]. Hepatic inflammation can be caused by viral infections; hepatotropic poisons, including industrial, plant, or fungal-derived poisons; and sterile stressors which are potentiated by metabolic changes that eventually manifest as acute and/or chronic liver diseases [3,4]. Although the purpose of hepatic inflammation is primarily to protect the liver from injury, excessive inflammation can cause irreversible damage to the liver parenchyma and compound the severity of various hepatic conditions. While all stages of liver diseases are associated with inflammation, the attenuation of pro-inflammatory pathways and enhancement of anti-inflammatories can reduce, and even stop, the progression of liver diseases. In contrast, hepatic inflammation can lead to fibrosis, cirrhosis, and, ultimately, hepatocellular carcinomas without proper treatment [2,5].

Bacterial lipopolysaccharides (LPS) have been found to be important cofactors in the pathogenesis of liver injury [6]. LPS are found within the walls of Gram-negative bacteria which induces toll-like receptor 4 (TLR4) signaling [2,7]. In NAFLD and alcoholic liver disease (ALD), genetics, diet, and/or alcohol induce gut barrier dysfunction, which alters intestinal permeability, causing endotoxemia [7,8]. As a result, LPS from the intestinal microflora induce TLR4 signaling [2]. As clinical manifestations of endotoxemia are largely due to LPS/TLR4 signaling, the administration of LPS in rodents provides a highly reproducible experimental model for studying endotoxemia, septic shock, and hepatic inflammation [9].

LPS-induced TLR4 signaling results in nuclear factor-κB (NF-κB) activation and relocation to induce transcription and the rapid release of inflammatory cytokines, such as tumor necrosis factor (TNF)-α, interleukin (IL)-1β, and IL-6, by neutrophils, macrophages, Kupffer cells, and other immune cells [10,11]. Cytokines, mitogens, and endotoxin can upregulate the rate-limiting enzyme cyclooxygenase 2 (COX-2) in immune cells to produce prostaglandins and thromboxane, which play a key role in generating the inflammatory response [12]. COX-2 expression and activity plays a crucial role in the initiation and progression of liver injury and fibrosis [12]. Produced primarily by macrophages, TNF-α is a pleiotropic cytokine which mediates septic shock in response to LPS and inflammatory mediators. TNF-α exerts its effects through two cognate membrane receptors known as TNF receptor (R)1 and 2. Within the liver, *TNF-α* plays an important role in regulating cell death, hepatocyte proliferation, and liver regeneration. The overproduction of TNF-α plays an important role in the pathophysiology of multiple liver diseases, including hepatitis and NAFLD [13]. IL-1β is a potent pro-inflammatory cytokine that initiates and magnifies a wide range of activities within the innate immune system through IL-1 receptor signaling [14]. The transcription of *IL-1β* has been shown to be the rate-limiting step of auto-inflammation and activate macrophages [15,16]. Importantly, the reduction in IL-1β in various diseases has been shown to reduce disease severity [15]. IL-6 is a pleiotropic cytokine that acts through a complex with glycoprotein 130 (gp130) to activate IL-6 receptors [17]. In the liver, IL-6 signaling regulates the processes of liver damage and regeneration while maintaining the balance between regulatory and effector T cells [17]. 

**Figure 1:**
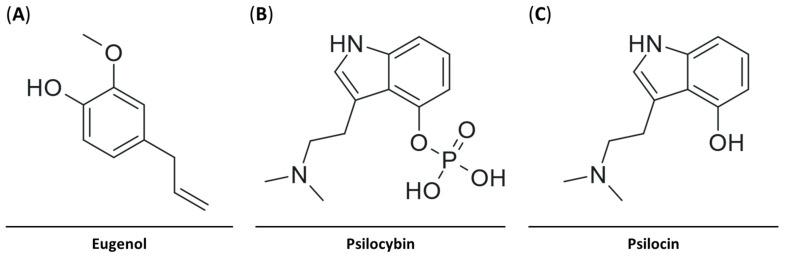
Two-dimensional representations of (**A**) eugenol, (**B**) psilocybin, and (**C**) psilocin, highlighting their structural variations and functional groups.

Eugenol (Figure 1), found in cloves, bay leaves, and allspice, has recently shown immense promise as a hepatoprotective antioxidant and anti-inflammatory therapeutic. Eugenol can alleviate liver damage by reversing cadmium-induced oxidative stress and pro-inflammatory cytokines [18]; reducing pro-inflammatory cytokine production induced by silver nanoparticles [19]; improving thioacetamide-induced hepatic injury by ameliorating the antioxidant status of the liver, which also reduces pro-inflammatory cytokines; and restoring liver function markers [20]. On the contrary, the anti-inflammatory properties of eugenol have not been tested in an LPS-induced model of hepatic inflammation.

Throughout history, psilocybin-containing mushrooms have been consumed due to their wide range of healing properties; however, it was not until recently that psilocybin was shown to have anti-inflammatory properties. Psilocybin’s active metabolite, psilocin, acts as a partial agonist on the serotonin receptor subtype 2A (5-HT2RA) (Figure 1). Although serotonin can display pro-inflammatory action through 5-HT2RA [21,22], psilocybin has been shown to have potent anti-inflammatory effects via 5-HT2RA on LPS-activated macrophages, resulting in the reduced expression of TNF-α, IL-1β, IL-6, and COX-2 in macrophages [23]. Even though psilocybin is known to have anti-inflammatory properties, its effect on liver inflammation has not yet been examined. Existing data on psilocybin’s effects on the liver center around its toxicity profile and show that psilocybin and psilocin are relatively nontoxic and physiologically well tolerated [20,24].

Previously, we have shown that psilocybin and eugenol can synergistically reduce COX-2 and IL-6 levels in human small intestinal epithelial cells treated with TNF-α/interferon-γ (IFN-γ) [25] and reduce TFN-α, IL-6, IL-8, IFN-γ, and granulocyte-macrophage colony-stimulating factor (GM-CSF) in human 3D EpiIntestinal tissue treated with TNF-α/IFN-γ [26], which suggest that psilocybin and eugenol could prevent gut–liver axis inflammatory diseases [27]. Furthermore, we have shown that psilocybin and eugenol can be used to treat LPS-induced brain inflammation in mice [28], as well as gut–brain axis-induced neuroinflammation induced via dextran sodium sulfate [29].

In this study, we recapitulated the LPS-induced liver inflammation model and subsequently tested the effects of psilocybin and/or eugenol on LPS-induced liver inflammation in mice. Given the previous studies reporting psilocybin and eugenol’s anti-inflammatory effects, we hypothesize that LPS-induced hepatic inflammation will be ameliorated by psilocybin and eugenol treatments, with the potential for synergistic interactions occurring between psilocybin and eugenol.

## 2. Results

### 2.1. Recapitulating an Inflammatory Mouse Model to Test Novel Anti-Inflammatory Compounds

Initially, we tested different end points following LPS injections to determine when cytokine production was upregulated in livers. We analyzed the levels of cytokines at 4 h, 24 h, and 48 h to analyze the time-dependent differences in cytokine production due to differences in immune cell recruitment and activation. At 4 h, neutrophils and Kupffer cells should be the primary cytokine producers within the liver. By 24 h, macrophages will be recruited and activated, whereas after 48 h circulating lymphocytes will be recruited and activated as well [30,31].

**Figure 2:**
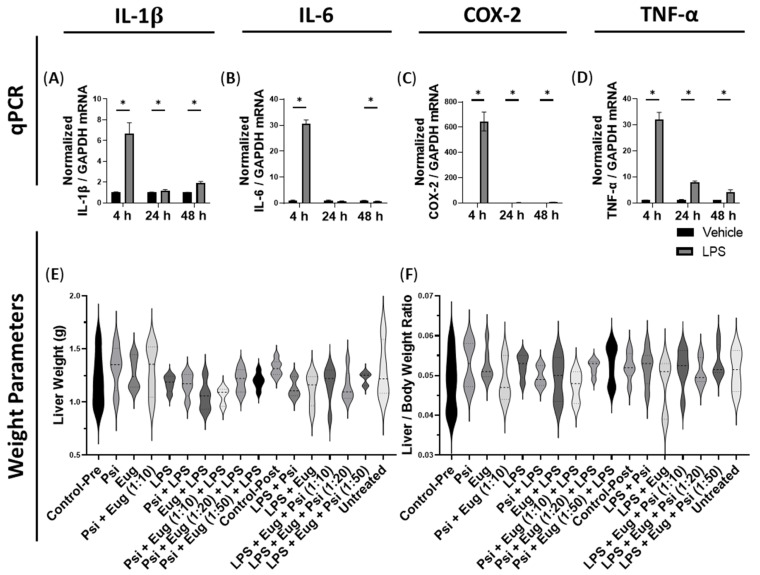
mRNA expression of pro-inflammatory cytokines within the liver 4, 24, or 48 h after LPS IP injection. These were measured via RT-qPCR for (**A**) *COX-2*, (**B**) *TNF-a*, (**C**) *IL-1β*, and (**D**) *IL-6* relative to *GADPH*, respectively. Data were analyzed with multiple unpaired Student’s *t*-tests with a false discovery rate correction (*Q* = 5%). Bars represent mean ± SEM (*n* = 3–6). Significance (*p*) is indicated by * *p* < 0.05 compared to the vehicle. (**E**) Violin plots indicate of the liver weight of mice orally fed with psilocybin and/or eugenol prior or after to LPS injection. (**F**) Violin plots indicate the ratio of the liver weight to body weight of mice treated with psilocybin and/or eugenol prior to or after LPS injection. Violin plots were analyzed with a one-way ANOVA followed by Tukey’s post hoc test. No significance was found.

We first measured *IL-1β*. It was found to be significantly higher 4 h (*p* < 0.05), 24 h (*p* < 0.05), and 48 h (*p* < 0.05) after LPS administration compared to the vehicle (Figure 2A).

Next, we measured *IL-6* transcript levels. We found that *IL-6* mRNA levels were upregulated after 4 h (*p* < 0.05), unchanged at 24 h (*p* = N.S.), and downregulated at 48 h (*p* < 0.05, Figure 2B).

We then measured *COX-2* mRNA levels. When treated with LPS, the mRNA expression of *COX-2* was significantly higher compared to controls after 4 h (*p* < 0.05), 24 h (*p* < 0.05), and 48 h (*p* < 0.05, Figure 2C).

Then, we measured *TNF-α* levels, which were found to be significantly upregulated compared to the controls 4 h (*p* < 0.05), 24 h (*p* < 0.05), and 48 h (*p* < 0.05, Figure 2D) after LPS administration. 

After recapitulating the LPS-induced liver inflammation model, we decided to test the anti-inflammatory properties of eugenol and/or psilocybin as both a pre-treatment and post-treatment relative to LPS administration (Figure in Section 4.1). An endpoint of 24 h after LPS administration was utilized to ensure macrophages were recruited and activated. In the pre-treatment groups, psilocybin and/or eugenol were administered orally via gavage twice prior to LPS IP injection (24 h and 48 h prior to LPS IP injection). In the post-treatment groups, psilocybin and/or eugenol were administered once orally via gavage 20 h after LPS IP injection and 4 h prior to tissue collection.

During mouse collections, the liver and body weight of the mice were recorded, while a ratio of their liver to body weight was calculated. We found no significant differences in liver weight (*p* = N.S., Figure 2E), but pre-treatment with eugenol appeared to lower the liver weight compared to the LPS group (*p* = N.S., Figure 2E). Similarly, the liver-to-body weight ratio was unaltered (*p* = N.S., Figure 2F).

### 2.2. Pre-Treatment and Post-Treatment with Psilocybin and/or Eugenol Decreases Cytokine mRNA Levels

Due to the previously reported anti-inflammatory effects of psilocybin and eugenol, we tested the effect of both compounds, when delivered orally both individually and together as a pre-treatment, on cytokine production 24 h after LPS administration.

**Figure 3:**
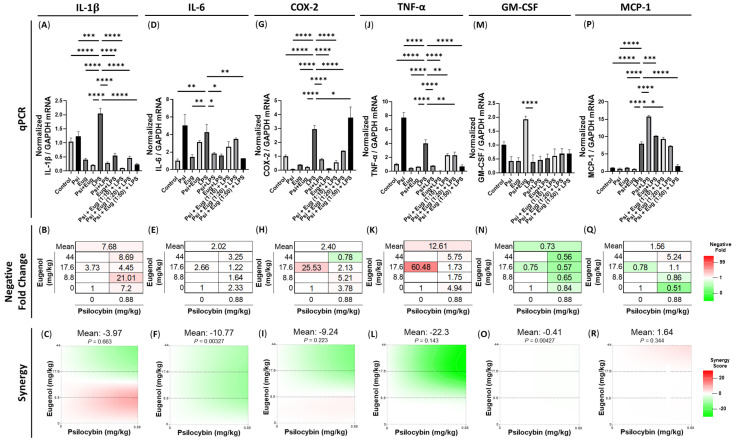
mRNA expression of pro-inflammatory cytokines within the liver with a pre-treatment of psilocybin, eugenol, or combinations of the two prior to LPS injection. Changes in mRNA expression were measured via RT-qPCR relative to *GAPDH*, the negative fold change was measured relative to LPS, and synergy was calculated for (**A**–**C**) *IL-1B*, (**D**–**F**) *IL-6*, (**G**–**I**) *COX-2*, (**J**–**L**) *TNF-α*, (**M**–**O**) *GM-CSF*, and (**P**–**R**) *MCP-1*. Data were analyzed with a one-way ANOVA followed by a Dunnett’s post hoc test and compared to the LPS group. Bars represent mean ± SEM (*n* = 3–6). Significance (*p*) compared to the LPS group is indicated within the figures using the following scale: * *p* < 0.05; ** *p* < 0.01; *** *p* < 0.001; **** *p* < 0.0001. The Loewe synergy score was calculated by SynergyFinder^+^.

LPS significantly upregulated *IL-1β* mRNA levels compared to the control (*p* < 0.0001, Figure 3A), while all treatment groups significantly lowered LPS-stimulated *IL-1β* mRNA levels (*p* < 0.0001, Figure 3A). Negative fold changes demonstrated the combination of 1:10 psilocybin and eugenol led to 21-fold lower *IL-1β* mRNA levels, normalized to LPS (Figure 3B). While the 1:20 combination of psilocybin and eugenol demonstrated potentiation, the average Loewe synergy score of this combination was −3.97 (*p* = N.S., Figure 3C) and combinations of 1:20 and 1:50 of psilocybin and eugenol resulted in antagonistic synergy.

Similarly, LPS upregulated *IL-6* mRNA levels compared to the control (*p* < 0.01, Figure 3D); however, psilocybin (*p* < 0.05), eugenol (*p* < 0.05), and the 1:50 combination of psilocybin and eugenol (*p* < 0.01) significantly downregulated LPS-stimulated *IL-6* mRNA levels compared to the LPS group (Figure 3D). The strongest decrease occurred with the 1:50 combination of psilocybin and eugenol, which resulted in a 3.25-fold decrease compared to the LPS group; eugenol alone induced a 2.66-fold decrease and psilocybin alone induced a 2.33-fold decrease in *IL*-6 levels normalized to the LPS group (Figure 3E). As a result, the mean Loewe synergy score of the 1:50 combination is −10.77 (*p* < 0.01, Figure 3F), indicating that psilocybin and eugenol have statistically significant antagonistic synergism.

*COX-2* mRNA levels were similarly upregulated by LPS compared to the control (*p* < 0.0001, Figure 3G). While both psilocybin (*p* < 0.0001) and eugenol (*p* < 0.0001) pre-treatments significantly lower *COX-2* levels, only the 1:10 and 1:20 combinations of psilocybin and eugenol significantly lower *COX-2* levels (*p* < 0.0001) compared to the LPS group (Figure 3G). In contrast, the 1:50 combination of psilocybin and eugenol significantly upregulated *COX-2* levels compared to the LPS group (*p* < 0.05, Figure 3G). Surprisingly, the eugenol pre-treatment resulted in the largest change, with a 25.5-fold decrease seen, whereas the combinations of psilocybin and eugenol demonstrated that larger doses of eugenol result in an inhibition of the decrease in *COX-2* levels (Figure 3H). Due to these results, little to no additive synergy is seen, with strong inhibition occurring with the 1:50 combination of psilocybin and eugenol and an overall mean Loewe synergy score of −9.24 (*p* = N.S., Figure 3I).

*TNF-α* transcript levels were also altered. Psilocybin (*p* < 0.0001), eugenol (*p* < 0.0001), 1:10 psilocybin and eugenol (*p* < 0.01), 1:20 psilocybin and eugenol (*p* < 0.01), and 1:50 psilocybin and eugenol (*p* < 0.0001, Figure 3J) significantly lowered LPS-stimulated *TNF-α* mRNA levels. Interestingly, the psilocybin treatment alone led to significantly higher *TNF-α* levels compared to the LPS group (*p* < 0.0001, Figure 3J).

To our surprise, LPS injections did not stimulate *GM-CSF* mRNA levels (*p* = N.S.); however, groups receiving pre-treatment with psilocybin and eugenol had significantly higher *GM-CSF* mRNA levels than the LPS group (*p* < 0.0001, Figure 3M). All pre-treatments resulted in a higher level of *GM-CSF* compared to the LPS group (Figure 3M), resulting in negative folds changes below 1 (Figure 3N). An overall Loewe synergy score of −0.41 (*p* < 0.01) indicates a slight, but significant, antagonism, which was uniform across all treatments (Figure 3O).

Lastly, *monocyte chemoattractant protein-1* (*MCP-1)*, an important chemokine that regulates monocyte and macrophage migration and infiltration [32], mRNA levels were significantly upregulated by LPS (*p* < 0.0001, Figure 3P). Interestingly, pre-treatment with psilocybin (*p* < 0.0001), eugenol (*p* < 0.001), and the 1:10 combination of psilocybin and eugenol (*p* < 0.05) resulted in significantly higher *MCP-1* levels compared to the LPS group (Figure 3P). However, the 1:50 combination of psilocybin and eugenol strongly downregulated *MCP-1* levels (*p* < 0.0001, Figure 3P) resulting in a 5.14-fold decrease in expression compared to the LPS group (Figure 3Q). As such, the overall mean Loewe synergy score was 1.56, with stronger potentiation occurring with more eugenol (*p* = N.S., Figure 3R).

After observing the results of pre-treatment with eugenol and psilocybin, we decided to test the efficacy of psilocybin and eugenol as a treatment post LPS injection. Treatments were administered orally 20 h after LPS injection, and then tissues were collected 4 h after gavage.

**Figure 4:**
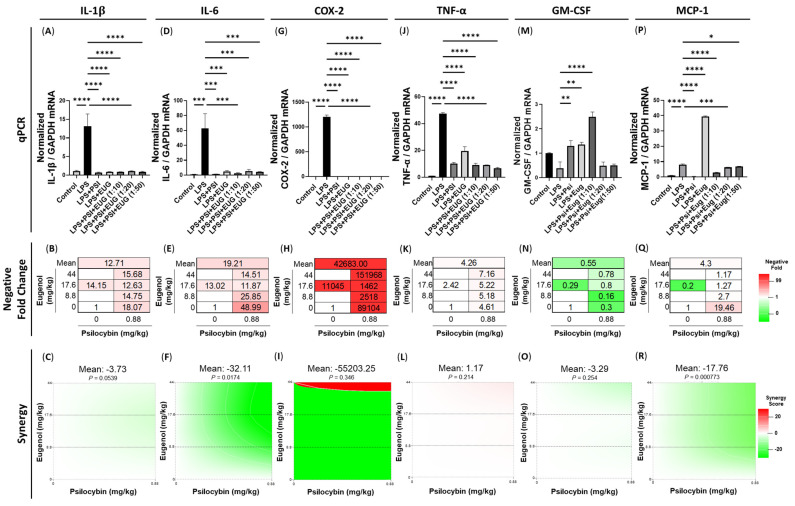
mRNA expression of pro-inflammatory cytokines within the liver with a post-treatment of psilocybin, eugenol, or combinations of the two following LPS injection. Changes in mRNA expression were measured via RT-qPCR relative to *GAPDH*, negative fold changes were measured relative to LPS, and synergy was calculated for (**A**–**C**) *IL-1B*, (**D**–**F**) *IL-6*, (**G**–**I**) *COX-2*, (**J**–**L**) *TNF-α*, (**M**–**O**) *GM-CSF*, and (**P**–**R**) *MCP-1*. Data were analyzed with a one-way ANOVA followed by a Dunnett’s post hoc test compared to the LPS group. Bars represent mean ± SEM (*n* = 3–6). Significance (*p*) compared to the LPS group is indicated within the figures using the following scale: * *p* < 0.05; ** *p* < 0.01; *** *p* < 0.001; **** *p* < 0.0001. The Loewe synergy score was calculated by SynergyFinder^+^.

LPS resulted in significantly upregulated *IL-1β* (*p* < 0.0001), *IL-6* (*p* < 0.001), *COX-2* (*p* < 0.0001), and *TNF-α* (*p* < 0.0001) mRNA levels compared to the vehicle, whereas all post-treatments significantly downregulated *IL-1β* (*p* < 0.0001), *IL-6* (*p* < 0.001), *COX-2* (*p* < 0.0001), and *TNF-α* (*p* < 0.0001) mRNA levels compared to the LPS (Figure 4). While all treatments downregulated *IL-1β* (Figure 4A), psilocybin alone produced the strongest decrease, with an 18.07-fold decrease generated (Figure 4B), while its Loewe synergy score of −3.73 (*p* = 0.0539, Figure 4C) suggests antagonism.

Similarly, all treatments downregulated *IL-6* levels (Figure 4D), but psilocybin provided the strongest decrease, with a 48.99-fold decrease seen (Figure 4E), and a statistically significant antagonistic synergism was present, with a mean Loewe score of −32.11 (*p* < 0.05, Figure 4F). While *COX-2* was downregulated by all treatments (Figure 4G), and all treatments resulted in a large fold decrease, the 1:50 combination of psilocybin and eugenol demonstrated the strongest fold decrease (Figure 4H). There was no significant synergism observed; however, the 1:50 combination demonstrated strong potentiation, while the 1:10 and 1:20 combinations of psilocybin and eugenol demonstrated antagonism (*p* = N.S., Figure 4I).

*TNF-α* demonstrated similar results, with a significant decrease seen with the post-treatments compared to the LPS (Figure 4J) and the 1:50 combination causing the largest change, with a 7.16-fold decrease (Figure 4K), and mild, but not significant, potentiation present, with a Loewe score of 1.17 (*p* = N.S., Figure 4L).

Similarly to the pre-treatments (Figure 4M), LPS resulted in a non-significant decrease in *GM-CSF* mRNA levels in the liver, while the post-treatment of psilocybin or eugenol (*p* < 0.01), and the 1:10 combination of psilocybin and eugenol (*p* < 0.001), significantly increased *GM-CSF* levels (Figure 4M). However, an increase in *GM-CSF* levels was not seen with either the 1:20 or 1:50 combinations of psilocybin and eugenol post-treatment (Figure 4M). No treatments decreased *GM-CSF* expression, and, in contrast, the 1:20 combination of psilocybin and eugenol had the lowest negative fold change of 0.16, which corresponds to a 6.25-fold increase in *GM-CSF* levels compared to LPS (Figure 4N). While the Loewe score suggested an antagonistic response, with an overall mean of −3.29, it was not significant (*p* = N.S., Figure 4O).

Lastly, *MCP-1* mRNA levels were upregulated by LPS (*p* < 0.0001) compared to the vehicle (Figure 4R). Interestingly, the eugenol post-treatment increased *MCP-1* levels (*p* < 0.0001), while psilocybin (*p* < 0.0001), as well as the 1:10 (*p* < 0.0001), 1:20 (*p* < 0.001), and 1:50 (*p* < 0.05) combinations of psilocybin and eugenol, significantly decreased *MCP-1* levels compared to LPS (Figure 4P). The psilocybin post-treatment resulted in the largest change, with a 19.46-fold decrease, while eugenol resulted in 5-fold increase in *MCP-1* levels normalized to the LPS group (Figure 4Q). The mean Loewe synergy score of −17.76 demonstrates that strong antagonism occurred, specifically at the higher doses of eugenol (*p* < 0.001, Figure 4R). As for *MCP-1*, eugenol’s stimulating effect on its mRNA levels appears to be mitigated by psilocybin in a dose-dependent manner (Figure 4P,O).

### 2.3. Post-Treatment with Psilocybin and/or Eugenol Decreases IL-12p70 Protein Levels

**Figure 5:**
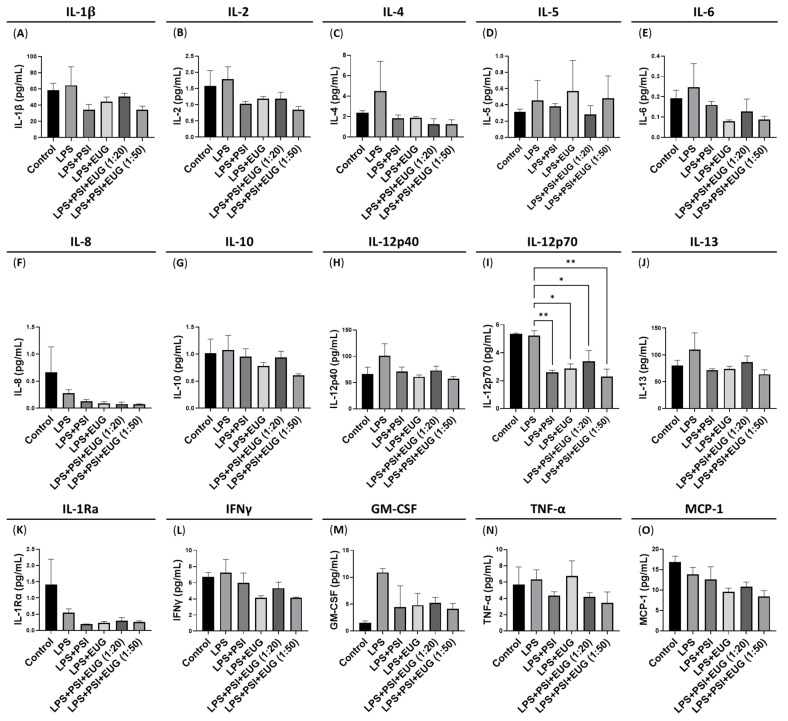
Levels of inflammatory cytokines after LPS intraperitoneal injections and oral psilocybin/eugenol post-treatment in liver tissue. The levels of (**A**) IL-1β, (**B**) IL-2, (**C**) IL-4, (**D**) IL-5, (**E**) IL-6, (**F**) IL-8, (**G**) IL-10, (**H**) IL-12p40, (**I**) IL-12p70, (**J**) IL-13, (**K**) IL-1Ra, (**L**) IFNγ, (**M**) GM-CSF, (**N**) TNF-α, and (**O**) MCP-1 were measured by ELISA. Data were analyzed with an ANOVA and Tukey’s post hoc test (*n* = 3). Replicates below the threshold were removed. Significance (*p*) is indicated within the figures using the following scale: * *p* < 0.05, and ** *p* < 0.01. Bars represent mean ± SEM.

Due to the efficacy of post-treatment with psilocybin and eugenol in downregulating the LPS-induced levels of pro-inflammatory genes (Figure 4), we quantified the levels of pro-inflammatory and anti-inflammatory cytokines secreted, including IL-1β (Figure 5A), IL-2 (Figure 5B), IL-4 (Figure 5C), IL-5 (Figure 5D), IL-6 (Figure 5E), IL-8 (Figure 5F), IL-10 (Figure 5G), IL-12p40 (Figure 5H), IL-12p70 (Figure 5I), IL-13 (Figure 5J), IL-1Ra (Figure 5K), IFNγ (Figure 5L), GM-CSF (Figure 5M), TNF-α (Figure 5N), and MCP-1 (Figure 5O).

While multiple cytokines demonstrated a trend of decreasing with a post-treatment of psilocybin and/or eugenol compared to the LPS group, these were not significant (*p* = N.S.). In contrast, only IL-12p70 protein levels were significantly downregulated by the post-treatment of psilocybin (*p* < 0.01), eugenol (*p* < 0.05), or the 1:20 (*p* < 0.05) and 1:50 combinations of psilocybin and eugenol (*p* < 0.01) compared to the LPS group (Figure 5I). However, LPS did not upregulate IL-12p70 protein levels compared to the control (*p* = N.S., Figure 5I). IL-12p70 is an active heterodimer composed of p35 and p40 which regulates innate responses through enhancing T helper 1, cytotoxic CD8+ T, and natural killer cell responses [33].

### 2.4. Pre-Treatment and Post-Treatment with Psilocybin and/or Eugenol Ameliorates LPS-Induced Histological Changes

**Figure 6:**
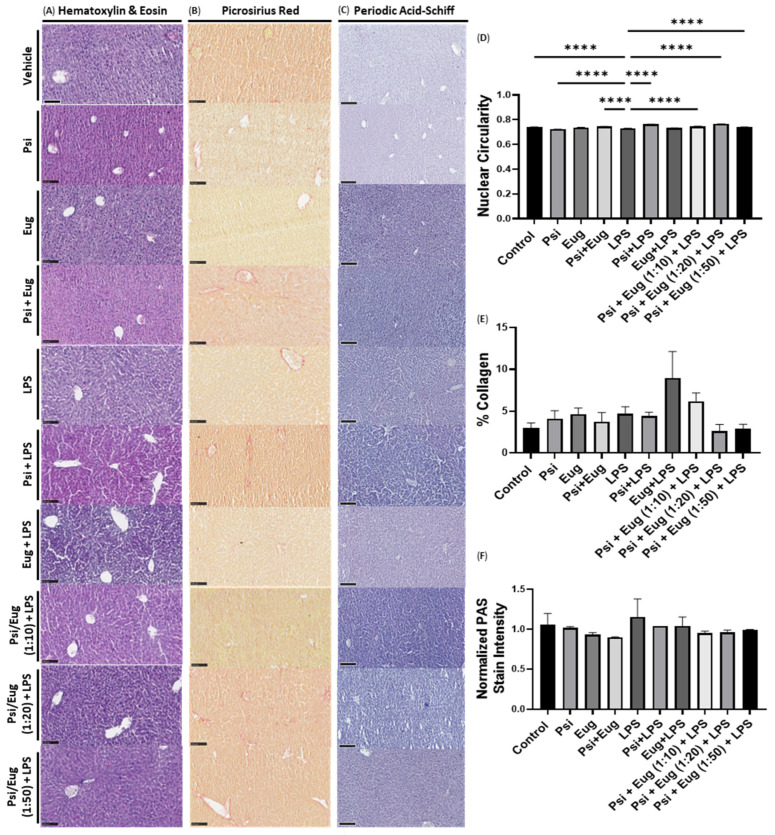
Histology of mouse livers pre-treated with psilocybin, eugenol, and combinations of the two following or before LPS injection. Sections of liver lobes were stained with (**A**) hematoxylin and eosin, (**B**) picrosirius red, and (**C**) periodic acid–Schiff. Scale bar = 100 µm. Stains were quantified by measuring (**D**) nuclear circularity, (**E**) the percentage stained, and (**F**) the intensity of the periodic acid–Schiff stain, normalized to the nuclei stain intensity. Data were analyzed with a one-way ANOVA followed by a Dunnett’s post hoc test compared to the LPS group or Tukey’s post hoc test. Bars represent mean ± SEM. (*n* = 3–6) Significance (*p*) compared to the LPS group is indicated within the figures using the following scale: **** *p* < 0.0001.

To assess liver structure, lobes were collected, sectioned, and stained. To assess the toxicity induced by either psilocybin or eugenol, we compared the psilocybin, eugenol, and psilocybin and eugenol treatments without LPS administration to the vehicle. We saw no noticeable toxic morphological changes in either the hematoxylin and eosin, picrosirius red, or periodic acid–Schiff (PAS) stain compared to the vehicle (Figure 6A–C).

Next, we compared the liver histology of LPS-exposed mice to that of the control. While we did find some morphological differences, the effects were minor. The histopathology of LPS-treated mice liver appeared to display an increase in inflammatory infiltration compared to the control, with the presence of cells that appeared to have altered nuclear shapes, with some nuclei appearing to have a clear internal phenotype, suggesting chromatin condensation for apoptosis. In contrast, there were no visible signs of hepatocyte necrosis, vacuolization and degeneration, lobule destruction, or Kupffer cell hyperplasia.

Compared to the LPS group, the pre-treatments of psilocybin and/or eugenol prior to LPS administration appeared to decrease the presence of inflammatory cell infiltration, lower the presence of pale pre-apoptotic nuclei, and change nuclear shapes. To quantify the nuclear shape, nuclear circularity was semi-automatically measured using QuPath. Compared to the control, LPS significantly decreased nuclear circularity (*p* < 0.0001, Figure 6D), whereas psilocybin and all combinations of psilocybin and eugenol ameliorated these effects (*p* < 0.0001), while eugenol did not (*p* = N.S., Figure 6D).

Next, we examined the histology of livers stained with picrosirius red to visualize the collagen content present to quantify changes in pro-fibrogenic responses. There were no significant changes noticed with any treatments; however, LPS did not increase the presence of collagen either (*p* = N.S., Figure 6E). Interestingly, eugenol pre-treatment appeared to increase collagen levels compared to LPS, but this was not significant (*p* = N.S., Figure 6E).

Lastly, we performed a PAS stain to measure glycogen content, which has previously been shown to be correlated with inflammation and is associated with select liver diseases. While we did not see any major or obvious differences between any groups, the glycogen content staining appeared to be higher in the LPS group, while combinations of psilocybin and eugenol appeared to ameliorate this effect (*p* = N.S., Figure 6F).

**Figure 7:**
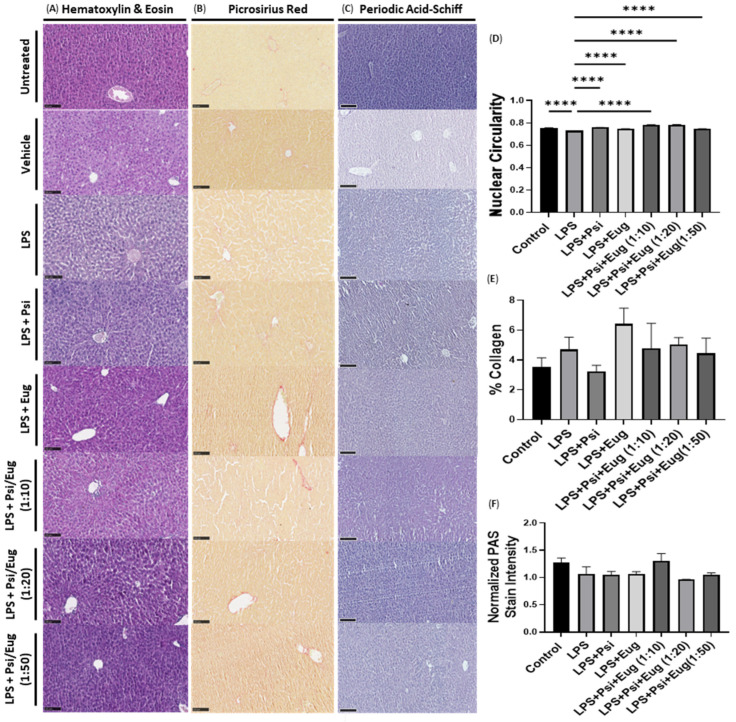
Histology of mouse livers post-treated with psilocybin, eugenol, and combinations of the two following or before LPS injection. Sections of liver lobes were stained with (**A**) hematoxylin and eosin, (**B**) picrosirius red, and (**C**) periodic acid–Schiff stains. Scale bar = 100 µm. Stains were quantified by measuring (**D**) nuclear circularity, (**E**) the percentage stained, and (**F**) the intensity of the periodic acid–Schiff’s stain, normalized to the nuclei stain intensity. Data were analyzed with a one-way ANOVA followed by a Dunnett’s post hoc test compared to the LPS group. Bars represent mean ± SEM. (*n* = 3–6) Significance (*p*) compared to the LPS group is indicated within the figures using the following scale: **** *p* < 0.0001.

Similarly to the pre-treatment, livers from mice treated with psilocybin and/or eugenol after LPS administration were collected, sectioned, and stained with hematoxylin and eosin (Figure 7A), picrosirius red (Figure 7B), and PAS reagent (Figure 7C). Just as seen in the pre-treatment groups, similar nuclear parameters were altered in the post-treatment groups, where there was a decreased presence of inflammatory cell infiltration and improved nuclear shape. Nuclear circularity was decreased in the LPS-exposed group (*p* < 0.0001), whereas it increased with all post-treatments (*p* < 0.0001, Figure 7D). Lastly, no changes (*p* = N.S.) were noticed in either collagen content (Figure 7E) or PAS stain intensity (Figure 7F) in the post-treatment groups compared to the LPS group.

## 3. Discussion

In this study, we aimed to investigate the anti-inflammatory effects of psilocybin and eugenol in liver inflammation. We recapitulated an LPS-induced inflammation model and were able to reliably induce inflammatory molecules within the liver, including upregulating the mRNA levels of *IL-1β*, *IL-6*, *COX-2*, and *TNF-α* (Figure 3 and Figure 4). Then, we tested whether either individual or combined oral treatments of psilocybin and eugenol at ratios of 1:10, 1:20, and 1:50, for either two daily pre-treatments twenty-four hours before LPS injection or one post-treatment twenty hours after LPS injection, could alter inflammatory mRNA levels, protein levels, and liver histology.

This study was performed because psilocybin has been suggested to be a potential therapeutic for reducing inflammation, even at sub-hallucinogenic doses, and has shown promise in cell and 3D tissues, as well as in the brain, in vivo. In addition to studying the effects of psilocybin, we tested the effects of eugenol on LPS-induced inflammation. Previous studies have suggested that eugenol may have a protective effect on liver inflammation [10,18,34,35], but the effects of eugenol on LPS-induced liver inflammation have never been studied. Lastly, we tested combinations of psilocybin and eugenol, as previous studies in vitro [25] have suggested a synergistic effect. If this effect is seen in vivo, a smaller dose of psilocybin and eugenol could be used.

As such, one of our main objectives was to test the efficacy of psilocybin and eugenol in reducing inflammation within the liver. In our model of inflammation, psilocybin reduced the mRNA levels of major pro-inflammatory proteins including *IL-1B*, *IL-6*, *COX-2*, and *TNF-α* (Figure 4 and Figure 5). Similarly, eugenol reduced the same pro-inflammatory proteins. As a pre-treatment, eugenol was more effective at reducing these major pro-inflammatory proteins, but psilocybin was more effective than eugenol as a post-treatment when compared to the LPS group (Figure 4 and Figure 5). The mRNA levels of these pro-inflammatory proteins were chosen due to their vast importance in liver inflammation.

IL-1β is a central component of acute and chronic inflammation. IL-1β is produced by activated macrophages as a proprotein and is proteolytically processed by caspase 1 within NLR family pyrin domain containing (NLRP)3 inflammasomes [36]. Once activated by caspase 1, IL-1β drives pro-inflammatory cytokine production, recruits immune cells, and modulates the effector actions of immune cells [16]. In our study, psilocybin decreased the expression of *IL-1β* mRNA when used as both a pre- and post-treatment. While the effect was about 5-fold stronger in the post-treatment group, there was a notable and significant decrease in the pre-treatment group. This could suggest macrophage recruitment or activation is affected by psilocybin. While no studies to date have shown whether psilocybin can affect macrophage recruitment, it is known that psilocybin can inhibit pro-inflammatory production in human macrophages [23], which is in line with the findings in our study.

IL-6 is an important pro-inflammatory cytokine that is primarily secreted by activated Kupffer cells during the acute phase of liver inflammation. IL-6 signals, through IL-6R, induce signal transducer and activator of transcription (STAT)3 signaling, which contributes to inflammation-induced liver injury [37]. Psilocybin significantly decreased *IL-6* levels in both pre- and post-treatment groups, which would suggest that psilocybin would likely act on Kupffer cells to inhibit *IL-6* production. Since this effect is more pronounced in the psilocybin post-treatment group compared to the pre-treatment group, psilocybin may assist in preventing the activation of Kupffer cells or other immune cells, but this would suggest that psilocybin is likely playing a larger role in altering the secretome of activated Kupffer cells.

COX-2 plays a complex and multifaceted role in liver function and disease. COX-2 is mainly expressed by non-parenchymal and inflammatory cells within the liver in response to stimuli, with large contributions from Kupffer cells, infiltrating macrophages, sinusoidal endothelial cells, and stellate cells. In normal liver tissue, COX-2 expression is typically low, but it can be rapidly induced in response to various stimuli during the initial stages of inflammation to protect the liver. However, COX-2 produces prostaglandins, which sensitize pain receptors, increase vasodilation and vascular permeability, and can exacerbate chronic inflammation. Since COX-2 can have both beneficial and harmful effects, considering the stage of liver damage and the source and quantity of COX-2 is important to decipher whether reducing COX-2 is beneficial. In our study, all post-treatment groups (Figure 4G) and all pre-treatment groups except the psilocybin and eugenol 1:50 group significantly downregulated *COX-2* levels compared to the LPS group (Figure 3G). The 1:50 combination of psilocybin and eugenol significantly increased *COX-2* levels instead (Figure 3G). Furthermore, this appeared to be a dose-dependent response, with increasing levels of *COX-2* seen as the eugenol concentration increased. Together, these facts suggest that both psilocybin and eugenol can act to reduce *COX-2* mRNA levels, likely decreasing prostaglandin production, while their effect is reversed in the 1:50 psilocybin–eugenol combination pre-treatment.

Within the immune system, TNF-α is a cytokine that acts upon TNF receptor (TNFR) 1 and 2. TNFR1 signaling is pro-inflammatory and apoptotic, while TNFR2 signaling is anti-inflammatory and promotes cell proliferation [38]. Additionally, the concentration of TNF-α determines its protective or damaging effect on the liver [39], with abnormally high levels playing a central role in liver inflammation [40], fibrosis [41], and damage [39]. TNF-α produced in the liver, primarily from Kupffer cells, infiltrating macrophages, T cells, hepatic cells, and sinusoidal endothelial cells, mediates and promotes pro-inflammatory responses that cause an excessive inflammatory response, resulting in liver damage. As both pre-treatment and post-treatment with psilocybin strongly downregulated *TNF-α* mRNA expression (Figure 3J and Figure 4J), this would suggest psilocybin would likely reduce damage to the liver.

The important multi-functional cytokine GM-CSF is a white blood cell growth factor secreted by macrophages, T cells, natural killer cells, and endothelial cells [42]. GM-CSF acts upon stem cells to produce granulocytes and macrophages. Within the liver, GM-CSF is produced by macrophages, acts as a chemoattractant of inflammatory cells, and regulates liver fibrosis [43,44]. We found that the LPS treatment did not induce an upregulation of *GM-CSF* levels (*p* = N.S.), while psilocybin and/or eugenol appeared to increase the levels of *GM-CSF* as a pre-treatment (*p* = N.S., Figure 3M) and each psilocybin and eugenol significantly increased mRNA levels as a post-treatment (*p* < 0.01, Figure 4M).

In addition, the change in the mRNA expression of the chemokine *MCP-1* was measured. MCP-1 is produced mainly by hepatocytes, Kupffer cells, hepatic stellate cells, and sinusoidal endothelial cells in the liver during the early stages of acute inflammation and is elevated in chronic liver diseases. MCP-1 is a potent chemoattractant that recruits monocytes to the liver, which then produce adhesion molecules and other pro-inflammatory cytokines. Importantly, *MCP-1* levels are strongly correlated with fibrosis, cirrhosis, and liver disease progression, and it is used as a marker of macrophage activation. Therefore, by measuring *MCP-1* levels, we can determine the effects of psilocybin on macrophage recruitment. While the psilocybin post-treatment significantly decreased *MCP-1* expression (Figure 4P), the psilocybin pre-treatment significantly increased *MCP-1* expression compared to the LPS group (Figure 3P). This suggests that if the liver were not in an inflammatory state, psilocybin could potentially induce monocyte infiltration and macrophage activation. In contrast, when in an inflammatory state, psilocybin can reduce macrophage recruitment. This suggests that using psilocybin microdoses for prolonged periods of time in individuals with healthy livers could potentially induce or exacerbate liver disease; however, the long-term effects of psilocybin microdosing on fibrosis, liver architecture, and cirrhosis in healthy individuals should be further studied before conclusions are made.

Although psilocybin reduced or appeared to reduce the mRNA of these pro-inflammatory proteins when used both as a pre-treatment and post-treatment, the size of its effect on mRNA downregulation when used as a post-treatment compared to the LPS group was much larger than when it was used as a pre-treatment (Figure 3 and Figure 4). This suggests that a post-treatment with psilocybin may be better for reducing inflammatory cytokines, chemokines, and other important regulators within the liver. In contrast, the psilocybin post-treatment might decrease the expression of inflammatory compounds beyond normal levels, resulting in negative effects downstream; however, this is unlikely.

Due to the strong downregulation of the mRNA of pro-inflammatory proteins during post-treatment with psilocybin/eugenol, we performed an ELISA on a comprehensive array of inflammatory proteins. This included IL-1β, IL-2, IL-4, IL-5, IL-6, IL-8, IL-10, IL-12p40, IL-12p70, IL-13, IL-1Rα, IFNγ, GM-CSF, TNF-α, and MCP-1 (Figure 5). Interestingly, multiple proteins, including IL-1β, IL-6, TNF-α, and MCP-1, were not significantly altered by LPS (Figure 5) but were significantly upregulated at the mRNA level (Figure 3 and Figure 4). Potentially, due to the short timeline between LPS administration and liver collection, there was not enough time to see significantly higher levels of pro-inflammatory proteins.

Importantly, IL-12p70 is an active heterodimer composed of p35 and p40 which regulates innate responses. IL-12p70 signaling enhances T helper 1, cytotoxic CD8+ T, and natural killer cell responses [33]. Furthermore, IL-12p70 is known to regulate granzyme B and perforin levels [45] in cytotoxic T lymphocytes and natural killer cells to induce inflammation-induced apoptosis, also known as pyroptosis [46,47]. Interestingly, IL-12p70 protein levels were significantly downregulated by psilocybin (Figure 5). This suggests that psilocybin’s anti-inflammatory effects may be due to it decreasing the IL-12p70-mediated activation of T helper 1, cytotoxic CD8+, and natural killer cell responses. 

During inflammation, the body can heal by removing damaged tissue and fighting infections. The effect of inflammation on healthy tissues can be detrimental if this response persists for a long time or becomes severe enough to cause irreparable damage. At the same time, hepatic inflammation, as with the inflammation of most organs, has a defensive role. During the last stage of inflammation, tissue damage can be repaired and/or regenerated, resulting in a restoration of homeostasis. It should be noted, however, that excessive or prolonged inflammatory responses are associated with a loss of hepatocytes, thereby causing irreversible damage to the liver parenchyma and compounding the severity of various hepatic conditions. Additionally, myofibroblasts produced by hepatic stem cells replace dead hepatocytes, and chronic inflammation can trigger fibrosis and cirrhosis and an irreversible decline in liver function [48]. While we did not see severe or prolonged inflammation, we decided to perform histology of the liver to assess the histopathological changes induced and complement our biochemical data.

We found a decrease in nuclear circularity (*p* < 0.0001, Figure 6D and Figure 7D) in LPS-treated mice compared to the control, which was ameliorated by psilocybin both pre-treatment and post-treatment (*p* < 0.0001, Figure 6D and Figure 7D). This suggests that the nuclear architecture may be altered after inflammation due to cytoskeletal remodeling, resulting in a deformed nucleus; chromatin condensation as a result of the activation or deactivation of genes during this response; DNA damage which disrupts the integrity of the nuclear envelope; or potentially apoptosis, resulting in nuclear blebbing or irregularities. While it is difficult to determine what changes downstream of inflammation resulted in altered nuclear circularity, psilocybin was able to reverse it. This would suggest that psilocybin is counteracting the negative effects of liver inflammation.

In addition, we performed a picrosirius red stain to examine collagen levels in the liver to examine the fibrogenic response within the liver. While LPS appeared to increase collagen levels, there was no significant difference compared to the control group (*p* = N.S., Figure 6E and Figure 7E). The administration of LPS and psilocybin pre-treatment did not appear to decrease the elevated collagen levels (*p* = N.S., Figure 6E); however, the administration of LPS and psilocybin post-treatment did appear to decrease elevated collage levels (*p* = N.S., Figure 7E). The decrease in collagen production induced by LPS could be related to psilocybin’s ability to strongly reduce pro-inflammatory protein. 

Lastly, we examined glycogen levels within the liver using a periodic acid–Schiff stain. Previous studies have shown that glycogen levels can be decreased in response to acute liver inflammation. In our study, we did not see any significant changes in glycogen content (*p* = N.S., Figure 6F and Figure 7F). It is likely that the short timeframe of 24 h after the intraperitoneal dose of LPS did not result in a liver response that was severe enough to be visualized in histological stains.

It is important to note that eugenol demonstrated properties that suggest it may have negative impacts on the liver. In particular, the eugenol pre-treatment increased the levels of *MCP-1* (Figure 3F), while, as a post-treatment, it increased the levels of both *GM-CSF* and *MCP-1* compared to the LPS group (Figure 4E,F). If this trend is seen at the protein level, this suggests that eugenol might increase the recruitment of monocytes/macrophages and stimulate the proliferation and maturation of multiple immune cell types, which would likely cause negative consequences. Furthermore, eugenol as a pre-treatment and as a post-treatment appeared to increase the levels of collagen within the liver, as demonstrated by the picrosirius red stain (*p* = N.S., Figure 6E and Figure 7E). While this trend was not significant, it is noteworthy. In contrast, when psilocybin and eugenol were administered simultaneously, these trends were not noticed. Therefore, it is possible that the addition of psilocybin can prevent the negative consequences of eugenol administration that were apparent in this study.

Due to the potential for psilocybin to offset the noted negative consequences of eugenol, the combination of psilocybin and eugenol would likely be a more suitable treatment for inflammation than eugenol alone. However, when compared to psilocybin alone, it is a bit unclear which is likely to lead to better outcomes. As a post-treatment, psilocybin alone led to the strongest reduction in IL-*1β*, *IL-6*, and *MCP-1* mRNA (Figure 4B,E,Q); however, the psilocybin eugenol co-treatment at a ratio of 1:50 led to a stronger reduction in *COX-2* and *TNF-α* mRNA levels (Figure 4H,K). In contrast, when comparing the effects of psilocybin alone and psilocybin and eugenol (1:50) at the protein level, there are no significant differences between the levels of the various pro-inflammatory proteins that were measured; however, the expressions of multiple proteins appear to be slightly lower in the combination treatment (Figure 5). Similarly, from the histology, the post-treatment of psilocybin alone and the co-treatment of psilocybin and eugenol at a 1:50 ratio appear to generate similar results; however, psilocybin alone appears to lead to lower collagen levels than the combination treatment (Figure 7E). These results suggest that either psilocybin alone or in combination with eugenol at a ratio of 1:50 as a post-treatment would likely have the best outcomes for liver inflammation in mice.

While the aim of this study was to determine the effects of psilocybin and eugenol on LPS-induced liver inflammation, we aimed to use this model to explore the potential effects of psilocybin and eugenol on acute and chronic liver inflammation. Liver inflammation is caused by LPS through TLR4 signaling. As a result of LPS activating TLR4, several pro-inflammatory, antiviral, and antibacterial cytokines are produced. TLR4 is expressed in several liver cells [7]. Due to its anatomical position, the liver is constantly exposed to gut-derived LPS in pathological circumstances. While the healthy liver has a low expression of TLR4, it possesses physiological mechanisms to suppress low-grade signaling. As a result, the healthy liver does not show signs of inflammation. A persistent elevation in inflammation cytokines may, however, occur as a result of increased exposure to LPS due to intestinal dysregulation and/or an increased TLR4 expression or sensitivity. This, in turn, can lead to chronic liver injury such as that seen in ALD, NAFLD, hepatocarcinoma, and fibrosis [7].

Our results are in line with previous studies in showing that eugenol has the potential to reduce liver inflammation. However, our study is the first to test eugenol as both a pre- and post-treatment in mice with LPS-induced liver inflammation. We have also shown that eugenol may have negative impacts on the liver, including increasing collagen production and even increasing *GM-CSF* and *MCP-1* levels. This may be a result of high doses of eugenol amplifying liver injury through oxidative and inflammatory mechanisms [49,50].

In contrast, research on the potential of psilocybin as an anti-inflammatory is nascent and has developed much more recently. In 2020, Nkadimeng et al. investigated the antioxidant and anti-inflammatory properties of *Psilocybe natalensis*, a psilocybin-containing mushroom, on LPS-stimulated RAW 264.7 macrophages [23] and found that it reduced LPS-induced NO and PGE2 production. This could suggest that the in vivo anti-inflammatory effects seen with psilocybin administration primarily occur due to the inhibition of a pro-inflammatory response from resident and recruited macrophages. Further research should inquire if and what effects are induced directly by psilocybin acting on parenchymal, stellate, and endothelial cells within the liver.

The observed anti-inflammatory effects of psilocybin are likely mediated by its ability to bind to various receptors in the gut and/or in the liver. After the consumption of psilocybin, four metabolites can be detected within the blood and urine: psilocin, 4-hydroxyindole-3-acetaldehyde (4-HIA), 4-hydroxyindole-3-acetic-acid (4-HIAA), and 4-hydroxytryptophan (4-HT), with the former being predominant [51]. Although psilocybin, a prodrug, can interact with receptors, psilocin is its main active metabolite. Psilocin interacts with multiple serotonin (5-hydroxytryptamine, 5-HT) receptors, including 5-HT_1A_ (0.123 μM) and 5-HT_2C_ (0.094 μM); however, its highest affinity is for 5-HT_2A_ (0.049 μM). Not surprisingly, most effects of psilocin are mediated through 5-HT_2A_ [52]. In addition, psilocin has a low binding affinity to other receptors, including adrenergic, dopaminergic, and other serotonergic receptors, at much higher concentrations [53]. The activation or inhibition of inflammation through the activation of serotonin receptors in part depends on whether psilocin preferentially binds receptors with an α_s_ or α_i_ subunit of the G protein [54]. The observed effects are likely a complex interplay of the activation of various receptors in cells associated with innate immunity responses.

Finally, it is important to note that we did not observe any evidence of hepatotoxicity with the tested doses of psilocybin. While generally it is believed that psilocybin mushrooms are not hepatotoxic, we found no conclusive evidence in the literature demonstrating hepatotoxicity or a lack of it in response to extracts of pure psilocybin.

## 4. Materials and Methods

### 4.1. Animal Handling, Exposure, and Harvesting

**Figure 8:**
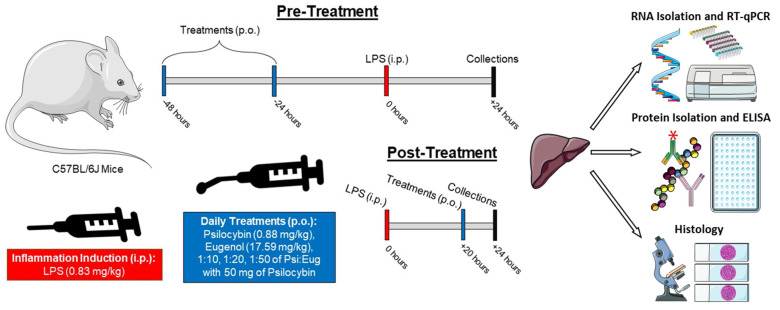
Timeline of the treatments of LPS, psilocybin, and eugenol administered in both pre- and post-treatment groups and the testing performed on excised livers. This figure was created using images from the Servier Medical Art library under a Creative Commons 4.0 license (http://smart.servier.com accessed on 20 February 2025).

This study used C57BL/6J mice (Charles River Laboratories, Laval, QC, Canada) in accordance with the Guide to Care and Use of Animals of the Canadian Council of Animal Care and was approved by the Animal Care Service at the University of Lethbridge (Protocol #2113, approved 25 April 2022). Mice were handled 14 days before beginning experimental treatments and tissue harvest to progressively train the mice. The mice were treated with psilocybin (0.88 mg/kg, CAS No. 520-52-50, Applied Pharmaceutical Innovation, Edmonton, AB, Canada), eugenol (17.59 mg/kg, CAS No. 97-53-0, Sigma-Aldrich, Saint Louis, MO, USA), or combinations of the two (1:10, 1:20, 1:50) via gavage (p.o.) either before (pre-treatment) or after (post-treatment) inter-peritoneal (i.p.) injections of LPS (0.83 mg/kg; LPS–L-4391-1MG, serotype 0111:B4 (Lot No. 059M4173V, SIGMA Life Science, Rehovot, Israel)) (Table 1). In the pre-treatment group, mice were gavaged two days prior and one day prior to LPS injection. In the post-treatment group, mice were gavaged 20 h after LPS injection. Twenty-four hours after LPS injection (4 h after gavage in the post-treatment group), mice were anesthetized with isoflurane and their tissues were collected (Figure 8). In addition, control mice with vehicle gavage treatments had their tissue collected 4, 24, and 48 h after LPS injection to identify the timepoints of cytokine production. Excised livers were washed in phosphate-buffered saline (PBS), briefly dried on a paper towel, and then weighed. One lobe was removed and processed for histology, while the remaining tissues were cut into small pieces, frozen in liquid nitrogen, and stored at −80 °C until utilized for molecular analysis. A timeline of the treatments and experimentation can be seen in Figure 8.

### 4.2. Protein Extraction and Quantification

Liver tissue protein was extracted using a trypsin (0.25%) and EDTA (2.21 mM) mixture (Cat# 325-043-EL, Wisent Inc., Saint-Jean-Baptiste, QC, Canada). The tissue mixture was centrifuged at 1600 rpm for 5 min. The supernatant was discarded, and the pellets were washed with ice-cold PBS twice. The pellet was dissolved in RIPA lysis buffer containing Tris-HCl (10 mM, pH 7.5), NaCl (100 mM), EDTA (1 mM), and PMSF (1 mM). The whole cellular protein lysate was sonicated using a Braunosonic model 1510 sonicator (B. Braun, Melsungen, Germany) at 80% operating capacity. The lysates were then centrifuged at 12,000× *g* for 10 min. The supernatant was removed, and the protein was quantified with the Bradford protein assay via a NanoDrop 2000/2000c Spectrophotometer (Thermo Fisher Scientific, Wilmington, DE, USA).

### 4.3. Multiplex Enzyme-Linked Immunosorbent Assay (ELISA)

Samples were snap-frozen in liquid nitrogen and stored at −80 °C until utilized. Three samples from each of the post-treatment groups were submitted to and processed by Eve Technologies (Calgary, AB, Canada) for the multiplexed quantification of cytokines, chemokines, and growth factors using Luminex xMAP technology. The multiplexing analysis was performed using the Luminex™ 200 system (Luminex, Austin, TX, USA).

### 4.4. RNA Extraction and Quantitative Real-Time PCR (RT-qPCR)

Total RNA was isolated from liver samples using the TRIzol^®^ Reagent (Invitrogen, Carlsbad, CA, USA) according to the manufacturer’s instructions. Samples were blended for 3 min and then incubated on ice for 3 min, and this was repeated three times. An additional 500 μL of TRIzol was added to the tubes and they were then incubated for 5 min. All samples were quantified using NanoDrop spectrometry (Thermo Fisher Scientific, Wilmington, DE, USA). Liver RNA was converted to cDNA using an iScriptTM Select cDNA synthesis kit (Cat# 1708897, BioRad, Hercules, CA, USA) according to the manufacturer’s instructions. Quantitative real-time PCR (qPCR) was performed with SsoFastTM EvaGreen^®^ Supermix (Cat# 1725202, BioRad, Hercules, CA, USA), and cDNA was generated from 500 ng of RNA. PCR reactions were based on 500 nM of forward and reverse primers that were specific to the target sequences of interest—the design of the primers came from the https://www.idtdna.com/Primerquest platform (accessed on 2 June 2022, Table 2)—and the SsoFastTIM EvaGreen^®^ Supermix (Cat# 1725202, BioRad, Hercules, CA, USA). The reference genes (GADPH) were analyzed using the GeNorm method, which involved a C1000TMR Thermo Cycler equipped with a CFX96 Touch^TM^ Real-Time PCR Detection System (BioRad, Hercules, CA, USA). A PCR was performed according to SsoFastTm guidelines, with the annealing temperatures as specified for the specific primer pairs used. BioRad Software (CFX Manager, V3.1) was used to perform the expression analysis and was based on the ΔΔCt method, using the reference genes that were stably expressed in the GeNorm Analysis. Each experiment included at least three biological replicates for each group.

### 4.5. Synergy Testing

The average fold differences calculated via the ΔΔCt method were used to calculate the average negative fold changes compared to the LPS group for each mRNA measured in each pre-treatment or post-treatment group receiving psilocybin and eugenol. Negative fold changes were inputted into R package SynergyFinder^+^ R-3.10.3 (SynergyFinder, Helsinki, Finland) to determine the Loewe synergy score for inhibition [55]. The imputation mode was not utilized. Two-dimensional heatmaps were created to visually show the differences in negative fold changes across treatments, with larger negative scores represented by a stronger red color. Two-dimensional maps were developed to demonstrate synergy scores across treatment combinations with a gradient from red, which demonstrates additive potentiation, to green, which demonstrates antagonism interactions.

### 4.6. Histology and Analysis

Liver lobes were fixed for 24 h in 4% paraformaldehyde, dehydrated in a series of ethanol dilutions, and cleared in xylene. Processed liver lobes were embedded in paraffin and serially sectioned at 5 μm. Sections were placed onto slides and stained, according to the manufacturers instructions, with either hematoxylin and eosin (Cat# NC1470670, Vector Laboratories, Newark, CA, USA), picrosirius red (Cat#: NC9908782. Polysciences Inc., Niles, IL, USA), or periodic acid–Schiff stain (Cat#: M1016460001, MilliporeSigma, Burlington, MA, USA). Each stain was analyzed using QuPath V0.5.1 (QuPath, Belfast, Northern Ireland). Nuclear circularity was calculated using the QuPath function on the hematoxylin and eosin stained slides with statistical analysis for each group using each nuclei measured as a separate replicate for statistical analysis. The percent collagen was calculated by using a colour threshold to determine the stained area of picrosirius red stained samples and dividing this by the area of liver tissue for each liver sample. Lastly, normalized PAS stain intensity was calculated by using a colour threshold to select the stained PAS stained area divided by the area of the liver tissue for each liver, which was then normalized to an untreated group. 

### 4.7. Statistical Analysis

The biological repeats (*n*) for each experiment are indicated in the figure captions. The results are presented as the mean of at least three samples per group, with a standard error of mean (SEM) or 95% confidence interval as indicated. Mean values, plus or minus the SEM, and statistical analyses were calculated and plotted using GraphPad Prism 9. The statistical analysis of data quantification was performed using a one-way ANOVA test and Dunnett’s post hoc test, or multiple unpaired Student’s *t*-tests with a false discovery rate correction (*Q* = 5%). Significance (*p*) was indicated within the figures using the following scale: *, *p* < 0.05; **, *p* < 0.01; ***, *p* < 0.001; ****, *p* < 0.0001.

## 5. Conclusions

Our data show that psilocybin can act as a potent anti-inflammatory and add to the previous studies demonstrating similar effects in vitro and in other organs. To our knowledge, this is the first study testing the effect of psilocybin on liver inflammation, and it builds on our previous studies showing that psilocybin and eugenol can act synergistically [25], reduce multiple pro-inflammatory proteins [26], and reduce in vivo brain inflammation induced through LPS and DSS [28,29]. However, this study suggests that psilocybin and eugenol may not create potentiation and could instead be antagonistic.

Based on this study, we demonstrate that psilocybin alone and the combination of eugenol and psilocybin reduce LPS-induced liver inflammation in mice, which suggests that psilocybin is a potential therapeutic of interest. Importantly, we did not notice any toxic effects of psilocybin on animals. Furthermore, psilocybin alone appears to have strong beneficial anti-inflammatory effects in vivo that justify further research into its potential as an anti-inflammatory therapeutic. In particular, treatment with psilocybin after the induction of inflammation demonstrated the best results, suggesting that future studies should be performed to study the efficacy of psilocybin and/or eugenol in treating human liver diseases. It would also be interesting to establish the concentration range for the potential hepatoxicity of psilocybin, as we found no such information in the literature.

In the future, it will be important to analyze the cytokines released by adaptive immunity Th1, Th2, and Th17 cells. For this, we would need to test the expression of relevant cytokines at later stages, from 48 h to several days after treatment.

## Figures and Tables

**Table 1:** Pre- and post-treatment groups and concentrations received of psilocybin, eugenol, or their combination, dissolved in 0.9% sodium chloride and administered via oral gavage before or after intraperitoneal LPS injection.

Pre-Treatments	Psilocybin (mg/kg)	Eugenol (mg/kg)
Control-Pre	0	0
Psi	0.88	0
Eug	0	17.59
Psi + Eug	0.88	8.8
LPS	N/A	N/A
Psi + LPS	0.88	0
Eug + LPS	0	17.59
Psi + Eug (1:10) + LPS	0.88	8.8
Psi + Eug (1:20) + LPS	0.88	17.6
Psi + Eug (1:50) + LPS	0.88	44.0
Control-Post	0	0
LPS + Psi	0.88	0
LPS + Eug	0	17.59
LPS + Psi + Eug (1:10)	0.88	8.8
LPS + Psi + Eug (1:20)	0.88	17.59
LPS + Psi + Eug (1:50)	0.88	44.0

Control-Post, Post-treatment vehicle; Control-Pre, Pre-treatment vehicle; Eug, eugenol; LPS, lipopolysaccharide; Psi, psilocybin.

**Table 2:** Primer sequences for each gene in the RT-qPCR analysis.

Target Gene	Forward Sequence (5′ → 3′)	Reverse Sequence (5′ → 3′)
*Il1b* Acc #: NM_008361.4	CAGGCAGGCAGTATCACTCATT	AAGAAGGTGCTCATGTCCTCATC
*Il6* Acc #: NM_001314054.1	GACTTCCATCCAGTTGCCTTCT	TATCCTCTGTGAAGTCTCCTCTCC
*Ptgs2* Acc #: NM_011198.4	CCTTCTCCAACCTCTCCTACTACA	AGCTCCTTATTTCCCTTCACACC
*Tnf* Acc #: NM_001278601.1	GCCTCTTCTCATTCCTGCTTGT	TGGGAACTTCTCATCCCTTTGG
*Csf2* Acc #: NM_009969(1)	AGCTCTGAATCCAGCTTCTC	CCACATCTCTTGGTCCCTTTA
*Ccl2* Acc #: NM_011333(1)	CTCGGACTGTGATGCCTTAAT	TGGATCCACACCTTGCATTTA
*Gapdh* Acc #: XM_036165840.1	CATCACTGCCACCCAGAAGA	AGTGGATGCAGGGATGATGTT

## Data Availability

Data are contained within the article and available upon request.
